# Prevalence and comorbidity of psychiatric disorders among 6-year-old children: 2004 Pelotas Birth Cohort

**DOI:** 10.1007/s00127-014-0826-z

**Published:** 2014-02-01

**Authors:** Sandra Petresco, Luciana Anselmi, Iná S. Santos, Aluísio J. D. Barros, Bacy Fleitlich-Bilyk, Fernando C. Barros, Alicia Matijasevich

**Affiliations:** 1 Post-Graduate Program in Epidemiology, Federal University of Pelotas, Rua Marechal Deodoro 1160, Pelotas, RS 96020-220 Brazil; 2 Mental Health Department, Federal University of Pelotas, Pelotas, Brazil; 3 Post-Graduate Program of Psychiatry, University of São Paulo, São Paulo, Brazil; 4 Post-Graduate Program in Health and Behavior, Catholic University of Pelotas, Pelotas, Brazil; 5 Department of Preventive Medicine, University of São Paulo, São Paulo, Brazil

**Keywords:** Prevalence, Cohort studies, Mental disorders, Mental health, Child

## Abstract

**Purpose:**

Most studies published on the prevalence of psychiatric disorders in children were conducted in high-income countries despite the fact that nearly 90 % of the world’s population aged under 18 live in low- and middle-income countries. The study aimed to assess the prevalence of psychiatric disorders among children of 6 years of age, to examine the distribution of psychiatric disorders by gender and socioeconomic status and to evaluate the occurrence of psychiatric comorbidities.

**Methods:**

The 2004 Pelotas Birth Cohort originally comprised 4,231 live births from Pelotas, southern Brazil. A total of 3,585 (84.7 % of 4,231 births) children aged 6 years were assessed using the Development and Well-Being Assessment (DAWBA).

**Results:**

Nearly 13 % of the children presented a psychiatric diagnosis according to DSM-IV, being more prevalent among males than females (14.7 and 11.7 %, respectively, *p* = 0.009). Anxiety disorders were the most prevalent of all disorders (8.8 %) and specific phobias (5.4 %) and separation anxiety disorder (3.2 %) were the most common subtypes. Attention deficit hyperactivity disorder (2.6 %), oppositional defiant disorder/conduct disorder (2.6 %), and depression (1.3 %) were also diagnosed. More than one psychiatric disorder was presented by 17 % of children. Socioeconomically disadvantaged children had a higher prevalence of psychiatric disorders.

**Conclusion:**

Our findings underline the early onset of psychiatric disorders among children and the frequent occurrence of psychiatric comorbidity. Early prevention is needed in the field of mental health in Brazil and should start during infancy.

## Introduction

Mental disorders and conditions account for as much as 30 % of the leading causes of loss of economic productivity and independence among adults worldwide [1]. Current studies have shown that around 50–75 % of mental disorders begin during childhood and adolescence [2, 3]. Mental disorders have also been associated with increased mortality in adult life [4]. Longitudinal studies have shown that mental disorders with onset occurring in childhood and adolescence are perpetuated throughout life greatly compromising normal development and having significant direct and indirect costs on society [3, 5–7].

There is a scarcity of epidemiological studies on mental disorders in children in low- and middle-income countries [6, 8–10]. A systematic review of population-based samples from low- and middle-income countries found that the prevalence of mental disorders varied between 10 and 20 % among children and adolescents [6]. Previous studies on the prevalence of mental disorders in childhood and adolescence conducted in Brazil using the Development and Well-Being Assessment (DAWBA) reported prevalence ranging from 7 to 13 % in different Brazilian regions [11–13], but none of them provided data on 6-year-old children.

The prevalence of psychiatric disorders in childhood and adolescence varies greatly depending on the instrument used, the age range studied, family characteristics, geographic location and socioeconomic status of the participants. Studies using the same diagnostic interview (DAWBA) found lower prevalence of psychiatric disorders in childhood and adolescence in high-income countries (7–9 %) than in countries of low and middle income (about 15 %) [14–17].

Population studies show that children and adolescents from underprivileged socioeconomic backgrounds have a higher prevalence of psychiatric disorders than those belonging to the higher socioeconomic classes [18–21]. The prevalence of different psychiatric diagnoses varies according to gender and age. Disruptive disorders and attention deficit and hyperactivity disorder are more prevalent in boys than in girls, while anxiety disorders are more prevalent in post-pubertal girls than in post-pubertal boys [18].

More than one psychiatric disorder with onset occurring in childhood and adolescence often co-occurs, making diagnosis difficult and further compromising the quality of life. In addition, individuals with psychiatric comorbidities might experience delay in diagnosis, which may aggravate their condition over time [22–24].

The present study aimed to assess the prevalence of psychiatric disorders among children aged 6 years, to examine the distribution of psychiatric disorders by gender and socioeconomic status and to evaluate the occurrence of psychiatric comorbidities. It adds to the group of studies on the prevalence and comorbidities of psychiatric disorders in children in low- and middle-income countries and provides input to policy planning in public health.

## Methods

### Setting and study design

The city of Pelotas is located in southern Brazil. It is a medium-sized city with 329,173 inhabitants and 205 inhabitants per square kilometer according to the 2010 Brazilian population census. Its population is predominantly urban (93.3 %). In spite of the economic improvement observed in the country in last 10 years, the Pelotas region had slower growth than the rest of the country. In 2010, Gross domestic product (GDP) per capita was US$ 5,976, lower than that observed for Brazil (US$ 8,161). However, the illiteracy rate in town for the year 2010 was considerably lower than for Brazil as a whole (4.1 vs. 8.7 %, respectively). The infant mortality rate for the city of Pelotas in 2011 was 15.1 per 1,000 live births, similar to that observed in Brazil (15.6 per 1,000 live births) [25–27].

A birth cohort study was conducted between January 1st and December 31, 2004 and included all births to mothers residing in the urban area of the city of Pelotas. In the city, more than 99 % of all deliveries take place in hospitals. Births were identified by daily visits to the city’s maternity hospitals. Of 4,287 births identified, 4,231 live births were evaluated and their mothers were interviewed within the first 24 h after delivery (refusal rate <1 %). A structured questionnaire was administered by trained interviewers to collect information about demographic, environmental and socioeconomic variables and on the characteristics of pregnancy, labor, delivery and health care service utilization.

The cohort children were followed up at several time points during their early life with high follow-up rates. The fifth follow-up was at the age of 6 years when a total of 3,585 children (84.7 % of the original cohort children) underwent a mental health assessment. Details of the study methods are published elsewhere [28, 29].

At the age of six, children and their mothers or caregivers were invited to attend a research clinic run by the Postgraduate Program of Epidemiology (Faculty of Medicine, Federal University of Pelotas, Brazil). Most interviews and assessments were carried out at the clinic but, when it was not feasible, they were conducted at the child’s home (19.0 %). These children are a heterogeneous group regarding their schooling, some children are in preschool and others have already begun the elementary cycle. In Brazil, only children who are aged six by March 31 may enroll in the first grade of elementary school, children who become 6 years old after this date must remain in preschool until the following year.

### Mental health assessment

All children were assessed using the DAWBA [30], an instrument that consists of a structured part and open-ended questions about symptoms of psychiatric disorders. The DAWBA is designed to generate psychiatric diagnoses according to International Classification of Diseases, 10th edition (ICD-10—World Health Organization, 1993) [31] and Diagnostic and Statistical Manual of Mental Disorders (DSM-IV**—**American Psychiatric Association, 1994) [32] criteria in children and adolescents age 5–17 years.

The DAWBA was administered to mothers or caregivers by trained interviewers, all of them are psychologists. Psychologists were trained through lessons about the functioning of DAWBA and psychopathology by both a child psychiatrist with over 10 years of clinical experience (SP) and a psychologist trained in conducting population-based studies (LA). Supervised interviews took place in the outpatient pediatrics and child psychiatric clinic of the Federal University of Pelotas.

The DAWBA combines highly structured questions based on diagnostic criteria from DSM-IV and ICD-10 with qualitative descriptions of any areas of difficulty. A computerized algorithm provides the probability of a child having any psychiatric disorder based on responses to structured questions, but clinical raters can review the symptoms, damage and qualitative information in parallel to these probabilities in their own assessment [33]. The clinical judgments were made by a child psychiatrist (SP), rater on this study, supervised by another child psychiatrist (BFB), who translated and validated the DAWBA for use in the Brazilian population [11].

For each evaluated psychiatric disorder, the interviewer asks about all symptoms, and other criteria required for a diagnosis according to DSM-IV and ICD-10. The time interval reported in the interview is the present and recent past. The interview incorporates “skip rules” that allow the interviewer to omit many of the questions in a section unless key screening questions are positive. When this is not the case, respondents are asked about all relevant symptoms of DSM-IV and ICD-10. Unless at least one of these symptoms is definitely present (or two symptoms in the hyperactivity section), the final interview questions about duration, start and impact of the symptoms are omitted. In the presence of positive symptoms in any area, respondents are surveyed with additional questions about the impact of these problems in the child’s life. These questions concern specific areas covering suffering and interference with family life, learning, friendships and leisure activities resulting from the symptoms. The information provided by structured questions on impact and symptoms is supplemented by open questions. If definite symptoms are identified by the structured questions, interviewers are instructed to use open questions and encourage respondents to describe the problems in their own words. These descriptions are transcribed verbatim by interviewers, but they are not evaluated by them [30].

Our version of DAWBA included sections for separation anxiety disorder, specific phobia, social phobia, generalized anxiety disorder, post-traumatic stress disorder, panic disorder and agoraphobia, obsessive–compulsive disorder, attention deficit hyperactivity disorder (ADHD), oppositional defiant disorder, conduct disorder, eating disorders, and tic disorder. In addition, five screening questions about development from a previous version of DAWBA were utilized. If any of these were positive, the open questions about development were also asked.

### Socioeconomic status (SES)

A wealth index (divided into quintiles) was used to measure SES. This index was constructed based on principal component analysis of ownership of consumer durable goods (e.g., TV, car, and computer) and household characteristics at birth (e.g., number of bathrooms, rental or owned home and use of housemaid services). The first principal component was used in the analysis [34].

### Statistical analyses

Descriptive analyses included estimation of frequency distributions for dichotomous variables with their related 95 % confidence intervals. The Chi square test and two-tailed tests were performed. All analyses were conducted for the entire sample and stratified by gender. Analyses were performed with Stata software version 12.1 (StataCorp LP, College Station, Tex).

The occurrence of psychiatric comorbidities was assessed in four major diagnostic groups: anxiety disorders, depressive disorders, ADHD and oppositional defiant disorder/conduct disorder. The diagnoses characterized by greater agitation and aggressiveness were then categorized into “externalizing disorders” which included ADHD/hyperkinetic disorders, oppositional defiant disorder and conduct disorder.

In the description of diagnostics, we use both DAWBA diagnostic classifications (DSM-IV and ICD-10) to facilitate comparison with other national and international studies that used only ICD-10 [16, 17], only DSM-IV [15, 18] or both diagnostic classifications [13, 14].

### Ethical issues

The study was approved by the Research Ethics Committee of the Federal University of Pelotas Medical School and has, therefore, been performed in accordance with the ethical standards laid down in the 1964 Declaration of Helsinki and its later amendments and in accordance with the Brazilian law. All mothers or caregivers signed an informed consent form that stated the purposes of the research studies and informed that they were free to decide whether or not to participate. All children who needed further assessment and advice based on the child psychiatrist’s evaluation were referred to local care services available in the area. Children whose mothers actively demanded it were referred for treatment, as were those children where the rater (SP) observed greater severity or risk and those in which the psychologists observed greater distress or impairment in day-to-day life. About 170 children were referred for any of the following services: outpatient child psychiatry at the Federal University of Pelotas, school assistance service of Pelotas, Child and Adolescent Psychosocial Care Center (CAPSI) and ambulatory psychological assessment at the Catholic University of Pelotas.

## Results

A total of 3,585 children were evaluated, of which 1,839 were male and 1,746 were female. The mean age was 6.8 years (SD 0.3 years). Missing information on psychiatric disorders was more common among children from the poorest families, birth weight <2,500 g at birth and gestational age <37 weeks of gestation. Children lost to follow-up were similar to those evaluated in terms of gender (15.2 vs. 15.4 %, boys and girls, respectively).

It was found that 13.2 % (*N* = 475) and 12.8 % (*N* = 458) of the children fulfilled criteria for at least one diagnosis of psychiatric disorder according to DSM-IV and ICD-10, respectively. Anxiety disorders were the most prevalent, present in nearly 9 % of all children. Specific phobia (5 %) and separation anxiety disorder (3 %) were the most prevalent anxiety disorders (Table 1). There were 46 cases of depression (1.3 %) according to both diagnostic criteria used. A higher prevalence of ADHD and hyperkinetic disorders were found according to DSM-IV (2.6 %) compared to ICD-10 (2.2 %), with predominance of ADHD-combined type (DSM-IV) and hyperkinetic disorder type (ICD-10). Oppositional defiant disorder (2 %) was more prevalent than conduct disorder (0.6 %) according to both DSM-IV and ICD-10 criteria. Autism, tic disorders, Gilles de la Tourette syndrome, eating disorders and stereotypies were rarely seen among children of age six (<1 %).

**Table 1:** Prevalence and confidence interval of different Psychiatric disorders among 6 years old, according to DSM-IV and ICD-10 diagnostic classifications

Psychiatric disorders	Prevalence DSM-IV		Prevalence ICD-10	
	*N*	% (CI 95 %)	*N*	% (CI 95 %)
Any diagnosis	475	13.2 (12.2; 14.4)	458	12.8 (11.7;13.9)
Any anxiety disorder	315	8.8 (7.9; 9.8)	308	8.6 (7.7; 9.6)
Separation anxiety disorder	113	3.2 (2.6; 3.8)	104	2.9 (2.4; 3.5)
Specific phobia	195	5.4 (4.7; 6.2)	192	5.4 (4.6; 6.1)
Social phobia	5	0.1 (0.05; 0.3)	5	0.1 (0.05; 0.3)
Agoraphobia	1	0.03 (0.001; 0.2)	1	0.03 (0.001; 0.2)
Post-traumatic stress disorder	27	0.8 (0.5; 1.1)	26	0.7 (0.5; 1.1)
Obsessive compulsive disorder	6	0.2 (0.1; 0.4)	6	0.2 (0.1; 0.4)
General anxiety disorder	6	0.2 (0.1; 0.4)	6	0.2 (0.1; 0.4)
Other anxiety	2	0.1 (0.001; 0.2)	2	0.1 (0.001; 0.2)
Any depressive disorder	46	1.3 (0.9; 1.7)	46	1.3 (0.9; 1.7)
Minor depression/mild	43	1.2 (0.9; 1.6)	19	0.5 (0.3; 0.8)
Major depression/severe	3	0.08 (0.02; 0.2)	3	0.08 (0.02; 0.2)
Dysthymia	0	−	0	−
Moderate	0		24	0.7 (0.4; 9.9)
Any undifferentiated anxiety or depression disorder	2	0.1 (0.001; 0.2)	2	0.1 (0.001; 0.2)
Any attention deficit hyperactivity disorder (ADHD)/Hyperkinetic disorders	93	2.6 (2.1; 3.2)	79	2.2 (1.7; 2.7)
ADHD combined	56	1.6 (1.2; 2.0)	−	−
ADHD inattentive	12	0.3 (0.2; 0.6)	−	−
ADHD hyperactive	13	0.4 (0.6; 1.9)	−	−
Other hyperactive disorder	12	0.3 (0.2; 0.6)	21	0.6 (0.4; 0.9)
Hyperkinetic disorder	−	−	58	1.6 (1.2; 2.1)
Any oppositional/conduct disorder	94	2.6 (2.1; 3.2)	91	2.5 (2.0; 3.1)
Oppositional defiant disorder	72	2.0 (1.6; 2.5)	70	2.0 (1.5; 2.5)
Conduct disorder	21	0.6 (3.5; 8.9)	20	0.6 (0.3; 0.9)
Other conduct disorder	2	0.1 (0.001; 0.2)	1	0.03 (0.001;0.2)
Autism^a^	10	0.3 (0.1; 0.5)	10	0.3 (0.1; 0.5)
Tic disorder and Tourette Syndrome	13	0.4 (0.2; 0.6)	13	0.4 (0.2; 0.6)
Eating disorders	1	0.03 (0.001; 0.2)	1	0.03 (0.001;0.2)
Stereotypies^a^	2	0.1 (0.001; 0.2)	2	0.1 (0.001; 0.2)

The 2004 Pelotas Birth Cohort (*N* = 3,585)

^a^Based on screening questions

### Gender and socioeconomic differences

Psychiatric disorders were more common among boys than girls (14.7 vs. 11.7 %). Male and female children showed very similar prevalence of depressive and anxiety disorders. ADHD was more prevalent among boys than girls (3.4 vs. 1.8 %) as were oppositional defiant/conduct disorders combined (3.7 vs. 1.5 %). Autistic spectrum disorders and tic disorders were also more commonly found in boys than girls, although this difference was not statistically significant (Table 2).

**Table 2:** Prevalence of various psychiatric disorder groups among 6 years old according to DSM-IV and ICD-10 diagnostic classifications and gender

Psychiatric disorders	DSM-IV				ICD-10			
	Boys *N* (%)	Girls *N* (%)	Chi square	*p* value	Boys *N* (%)	Girls *N* (%)	Chi square	*p* value
Any diagnosis	270 (14.7)	205 (11.7)	6.734	0.009	260 (14.1)	198 (11.3)	6.292	0.012
Any anxiety disorder	162 (8.8)	153 (8.8)	0.002	0.961	155 (8.4)	153 (8.8)	0.128	0.721
Any depressive disorder	27 (1.5)	19 (1.1)	1.021	0.312	27 (1.5)	19 (1.1)	1.021	0.312
Any ADHD disorder/hyperkinetic	62 (3.4)	31 (1.8)	9.028	0.003	56 (3.1)	23 (1.3)	12.407	<0.001
Any oppositional/conduct disorder	68 (3.7)	26 (1.5)	17.110	<0.001	66 (3.6)	25 (1.4)	16.845	<0.001
Autism	8 (0.4)	2 (0.1)	3.307	0.069	8 (0.4)	2 (0.1)	3.307	0.069
Tic disorder and Tourette Syndrome	9 (0.5)	4 (0.2)	1.680	0.195	9 (0.5)	4 (0.2)	1.680	0.195

The 2004 Pelotas Birth Cohort (*N* = 3,585)

*ADHD* Attention deficit hyperactivity disorder

Children from lower-income families had a higher prevalence of any mental disorder than those from higher-income families (14 vs. 8 %). The prevalence of ADHD and oppositional/conduct disorder was significantly different across SES categories, but the same was not seen for depression and anxiety disorders (Table 3).

**Table 3:** Prevalence (*N*) of various psychiatric disorder groups among 6 years old according to DSM-IV and ICD-10 diagnostic classifications and socioeconomic status at birth

Psychiatric disorders	Prevalence of psychiatric diagnoses by DSM-IV							Prevalence of psychiatric diagnoses by ICD-10						
	Wealth index, quintiles					Chi square	*p* value	Wealth index, quintiles					Chi square	*p* value
	1st	2nd	3rd	4th	5th			1st	2nd	3rd	4th	5th		
Any diagnosis	14.7 (98)	14.7 (111)	15.1 (108)	13.6 (101)	8.1 (57)	20.583	<0.001	14.1 (94)	14.3 (108)	14.4 (103)	13.0 (97)	8.0 (56)	18.611	0.001
Any anxiety disorder	9.9 (66)	9.3 (70)	9.9 (71)	8.2 (61)	6.7 (47)	6.440	0.169	9.2 (61)	9.1 (69)	10.0 (72)	8.1 (60)	6.6 (46)	6.379	0.173
Any depressive disorder	1.1 (7)	1.9 (14)	1.8 (13)	1.2 (9)	0.4 (3)	7.889	0.096	1.1 (7)	1.9 (14)	1.8 (13)	1.2 (9)	0.4 (3)	7.889	0.096
Any ADHD disorders/hyperkinetic	2.6 (17)	2.9 (22)	4.5 (32)	2.3 (17)	0.7 (5)	20.294	<0.001	2.7 (18)	2.5 (19)	3.2 (23)	1.9 (14)	0.7 (5)	12.022	0.017
Any oppositional/conduct disorder	3.0 (20)	4.0 (30)	2.9 (21)	2.6 (19)	0.6 (4)	17.574	0.001	2.7 (18)	3.8 (29)	2.9 (21)	2.6 (19)	0.6 (4)	16.633	0.002

The 2004 Pelotas Birth Cohort (*N* = 3,585)

*ADHD* attention deficit hyperactivity disorder

### Comorbidities

Among children with psychiatric disorders, a single diagnosis was made in 393 (82.7 %) and 383 children (83.6 %) according to DSM-IV and ICD-10 criteria, respectively. The occurrence of more than one diagnosis was seen among 82 (17.3 %) and 75 (16.4 %) children according to DSM-IV and ICD-10 criteria, respectively. Among children with more than one diagnosis, one psychiatric comorbidity was seen in 67 children (81.7 %) by DSM-IV and 60 children (80 %) by ICD-10 criteria. Two or more comorbidities were seen in 15 children according to both diagnostic criteria.

Comorbidities were analyzed in major groups of disorders. The most common comorbidities according to ICD-10 and DSM-IV criteria were: hyperkinetic disorder and oppositional/conduct disorder (24 children, 29 %); anxiety disorder and depression (15 children, 18 %); and anxiety disorder and oppositional/conduct disorder (13 children, 16 %). Two psychiatric comorbidities were seen in 12 children, while three comorbidities were seen in only three children, all of them males.

## Discussion

The present study evaluated psychiatric disorders among 6-year-old children from the 2004 Pelotas Birth Cohort. It was found that nearly 13 % of the cohort children fulfilled criteria for diagnosis of at least one psychiatric diagnosis according to either DSM-IV or ICD-10. The prevalence of any psychiatric diagnosis was higher among boys, mainly due to externalizing disorders. Children from lower-income families had a higher prevalence of any mental disorder than those from higher-income families. Psychiatric comorbidities were seen in 17 % of children with a psychiatric diagnosis (DSM-IV); more than one externalizing disorders were the most commonly associated conditions.

The prevalence of psychiatric disorders found in this study is in an intermediate position between the prevalence observed in low-income and high-income countries. Considering only studies that used DAWBA as a diagnostic tool, the prevalence of psychiatric disorders in high-income countries ranged from 7 % (95 % CI 5.6; 8.5) in Norway [14] to 7.8 % in Great Britain [15]. The Norwegian study evaluated a sub-sample of 1,011 children aged 8–10 years and the British study investigated a sample of 2,964 children aged 5–7 years. The prevalence of psychiatric disorders reported among children and adolescents aged 5–14 years from middle-income countries was around 15 %. The prevalence of psychiatric disorders was 15.7 % (95 % CI 11.7; 20.2) in Yemen [35], 15.2 % (95 % CI 10.9; 20.8) in Bangladesh [17], and 15.3 % (95 % CI 10.4; 20.1) in Russia [16].

Fleitlich-Bilyk and Goodman evaluated a sample of 1,251 schoolchildren and adolescents aged 7–14 from an urban area of a city in southeast Brazil with DAWBA and reported a prevalence of 12.7 % (95 % CI 9.8; 15.5) of any psychiatric disorder according to DSM-IV criteria [11]. Another study conducted in a rural area of the northeastern state of Bahia, Brazil, assessed 430 children and adolescents age 7–14 years with SDQ in the first phase and 100 children with DAWBA in the second phase and found a prevalence of psychiatric disorders of 7 % (95 % CI 2.3; 11.8) [12]. The authors argued that the low prevalence of psychiatric disorders found in their study in Bahia was probably due to the fact that parents underreported the impact of symptoms on their children’s lives. However, the small sample size of the study could have explained at least in part their results.

In the present study, anxiety disorders were the most prevalent of all psychiatric disorders (8.8 %). It is possible that the high prevalence of anxiety disorders could be explained by the age of the children assessed, which is consistent with the age of onset of some anxiety disorders. Studies investigating the age of onset of psychiatric disorders showed that specific phobia and separation anxiety disorders are conditions that appear early in childhood at a mean age of 7 years and that 50 % of separation anxiety and specific phobia cases usually occur before the ages of 5 and 8 years, respectively [36, 37]. In the US, the National Comorbidity Survey-Adolescent Supplement (NCS-A) investigated a sample of 10,123 adolescents and reported that the mean age of onset of separation anxiety disorders and specific phobia was 6 years [38]. The prevalence of anxiety disorders varies throughout child development. Separation anxiety disorders usually have an early onset, but there is a gradual reduction in its prevalence after the age of 10 years [37]. Thus, the high prevalence of separation anxiety disorders and specific phobia found in our study may be explained by the fact that almost all children assessed were either 6 or 7 years old, an age when these conditions are most common.

Concerning ADHD prevalence, previous reports from other Brazilian settings conducted using DAWBA showed lower prevalence than in the present study (1.8 and 0.9 % in southeast and northeast regions of Brazil, respectively) [11, 12]. However, all of these prevalences are lower than the 5 % worldwide prevalence of ADHD reported in a meta-analysis by Polanczyk et al. [39] which included studies published between 1978 and 2005. The finding of a lower prevalence of ADHD in the present study may be due to the age of the cohort assessed. In Brazil, children at age 6 and 7 years are starting their school life and do not have a history of school problems or failure. Attention problems, hyperactivity and learning difficulties are usually first noticed by teachers rather than by parents because of the structured school setting. Furthermore, the fact that we did not administer the DAWBA to teachers may have caused a reduction in the reporting of ADHD symptoms and, consequently, the rate of diagnosis [15, 38]. It should be noted that current diagnostic criteria for ADHD are being reviewed as it now seems that the disease may in some cases have a later onset between 7 and 12 years [40, 41]. The Brazilian cohort study conducted with adolescents aged 11 years in Pelotas found a 4.1 % prevalence of ADHD [13].

Angold et al. [42] evaluated the prevalence generated by three different styles of psychiatric interviews, those based on respondents (Diagnostic Interview Schedule for Children, DISC), those based on the interviewers (Child and Adolescent Psychiatric Assessment, CAPA) and interviews based on “expert judgment” (DAWBA) applying them to the same sample of children and adolescents aged 9–16 years. The authors found that 17.7 % of young people had one or more diagnoses with DAWBA, 47.1 % with DISC and 32.4 % with CAPA (excess of DISC diagnoses occurred due to specific phobias). The authors found that DAWBA detected more severe cases. Agreement of the three instruments was lower in anxiety disorders and DAWBA generated significantly fewer cases of depression and anxiety than CAPA. Similar rates were found in behavioral disorders (attention deficit and hyperactivity disorders, oppositional defiant disorders and conduct disorders) for all three instruments. Thus, the prevalence of different psychiatric disorders observed in our study could be higher if we had used other diagnostic interview.

Boys showed a higher prevalence of psychiatric disorders than girls, which is consistent with what was reported in the British Child and Adolescent Mental Health Survey of 1999 (BCAMHS-99) among children aged 5–15 years [15]. Boys also had significantly more externalizing disorders (ADHD and oppositional/conduct disorders) than girls, a finding that is in agreement with what has been reported in many other international studies. A US study of lifetime ADHD prevalence showed a ratio of boys to girls of 2.28 to 1 [43]. A Brazilian study conducted in the southeastern region observed higher rates of externalizing disorders among boys than girls: 2.7 vs. 0.7 % for any ADHD and 10.0 vs. 3.5 % for any oppositional/conduct disorder [11]. The same was seen in the BCAMHS-99: 3.6 vs. 0.9 % for any ADHD and 3.2 vs. 1.4 % for oppositional defiant disorders among boys and girls, respectively [15].

Socioeconomically disadvantaged children had a higher prevalence of psychiatric disorders than those from better-off families, a finding that is consistent with a large number of previous studies [21, 44, 45] including an epidemiological survey that assessed 898 children in the southeast of Brazil [46]. In our study, only externalizing disorders (ADHD and oppositional defiant/conduct disorder) were more frequent among the poorest children; no significant difference was found in the prevalence of internalizing disorders (anxiety and depressive disorders) between children of poor and better-off families. Costello et al. [19] found a lower rate of behavioral symptoms (oppositional defiant and conduct disorder) in children of parents with higher income, but there was no difference regarding emotional symptoms (anxiety and depression).

The prevalence of children who had one or more psychiatric comorbidities in our study is very similar to those observed in southeastern Brazil (21 %), England (22 %) and the United States of America (20 %) [11, 15, 38]. The most common comorbidities identified in our study (ADHD and oppositional defiant/conduct disorder; depression and anxiety disorders) were the same as those found in the previous cited studies [11, 15, 38]. A longitudinal study “The Great Smoky Mountains Study” reported a significant increase in comorbidities with age [18]. Egger and Angold [47] reviewed studies with preschoolers and found they had similar rates of psychiatric disorders and common comorbidities to those observed in older children. The rate of comorbidities at an early age found in the present study has major clinical implications for mental health providers who should be aware of the occurrence of comorbidities as well as of the possibility of a very early onset of psychiatric disorders in preschool children.

### Strengths and limitations of the study

The present study has several strengths: a large population-based sample of children assessed using an internationally recognized instrument designed to generate diagnosis of psychiatric disorders, administered by highly trained psychologists that ensured good quality data. However, some methodological difficulties of the study need to be discussed. First, the study relied only on mothers or caregivers as informants, as we did not apply the DAWBA version for teachers. Second, as we administered only the screening questions of the DAWBA development section, our ability to assess the prevalence of autistic spectrum disorders was limited. Third, the prevalence of psychiatric disorders we found may be slightly underrated because subjects who were not able to follow-up and were, therefore, not shown in the cohort evaluation tended to have impoverished socioeconomic status, prematurity and/or low birth weight, all of these are known risk factors for psychiatric disorders. Finally, in our study we were not able to perform interrater reliabilities. However, our rater (SP) undertook the online training from the DAWBA webpage and subsequently was personally trained by Bilyk-Fleitlich who was personally and extensively trained by Goodman, author of DAWBA [30]. The diagnostic reliability between Fleitlich-Bilyk and Goodman showed kappa value of 0.93 for any disorder, 0.91 for any emotional disorder, 1.00 for any ADHD and 1.00 for any oppositional/conduct disorder. Bilyk-Fleitlich has been the rater in all Brazilian research using DAWBA, including the study carried out on the island of Maré, Bahia, by Goodman et al. [12] and research with adolescents from Pelotas made by Anselmi et al. [13].

## Conclusions

The present study found a prevalence of psychiatric disorders and comorbidities among children aged six which were similar to previous studies, both Brazilian and international ones. However, none of the previous Brazilian studies assessed 6 years old or displayed a full psychiatric assessment of the whole sample. Our findings are important as they underline the early onset of psychiatric disorders in children, especially anxiety disorders, and the occurrence of psychiatric comorbidities. This information should be used in service planning and by policy makers to provide better conditions to meet the mental health needs of Brazilian children. Moreover, our results point out that detection is needed in the field of mental health in Brazil and should start during infancy.

## References

[1] Lancet Global Mental Health Group, Chisholm D, Flisher AJ, Lund C, Patel V, Saxena S, Thornicroft G, Tomlinson M (2007). Global mental health 6. Scale up services for mental disorders: a call for action. Lancet.

[2] Kessler RC, Berglund P, Demler O, Jin R, Merikangas KR, Walters EE (2005). Lifetime prevalence and age-of-onset distributions of dsm-iv disorders in the national comorbidity survey replication. Arch Gen Psychiatry.

[3] Kessler RC, Amminger GP, Aguilar-Gaxiola S, Alonso J, Lee S, Ustun TB (2007). Age of onset of mental disorders: a review of recent literature. Curr Opin Psychiatry.

[4] Gale CR, Batty GD, Osborn DP, Tynelius P, Whitley E, Rasmussen F (2012). Association of mental disorders in early adult life and later psychiatric hospital admissions and mortality in a cohort study of more than 1 million men. Arch Gen Psychiatry.

[5] Petrou S, Johnson S, Wolke D, Hollis C, Kochhar P, Marlow N (2010). Economic costs and preference-based health related quality of life outcomes associated with childhood psychiatric disorders. Br J Psychiatry.

[6] Kieling C, Baker-Henningham H, Belfer M, Conti G, Ertem I, Omigbodun O, Rohde LA, Srinath S, Ulkuer N, Rahman A (2011). Child and adolescent mental health worldwide: evidence for action. Lancet.

[7] de Girolamo G, Dagani J, Purcell R, Cocchi A, McGorry PD (2012). Age of onset of mental disorders and use of mental health services: needs, opportunities and obstacles. Epidemiol Psychiatr Sci.

[8] Duarte C, Hoven C, Berganza C, Bordin I, Bird H, Miranda CT (2003). Child mental health in Latin America: present and future epidemiologic research. Int J Psychiatry Med.

[9] Belfer ML (2008). Child and adolescent mental disorders: the magnitude of the problem across the globe. J Child Psychol Psychiatry.

[10] Rohde LA (2011). The need of epidemiological data on child mental disorders from low-middle income countries. Eur Child Adolesc Psychiatry.

[11] Fleitlich-Bilyk B, Goodman R (2004). The prevalence of child psychiatric disorders in southeast Brazil. J Am Acad Child Adolesc Psychiatry.

[12] Goodman R, Neves dos Santos D, Robatto Nunes AP, Pereira de Miranda D, Fleitlich-Bilyk B, Almeida Filho N (2005). The Ilha de Maré study: a survey of child mental health problems in a predominantly African-Brazilian rural community. Soc Psychiatry Psychiatr Epidemiol.

[13] Anselmi L, Fleitlich-Bilyk B, Menezes AM, Araújo CL, Rohde LA (2010). Prevalence of psychiatric disorders in a Brazilian birth cohort of 11-year-olds. Soc Psychiatry Psychiatr Epidemiol.

[14] Heiervang E, Stormark KM, Lundervold AJ, Heimann N, Goodman R, Posserud MB, Ullebø AK, Plessen KJ, Bjelland I, Lie SA, Gillberg C (2007). Psychiatric disorders in Norwegian 8- to 10-year-olds: an epidemiological survey of prevalence, risk factors, and service use. J Am Acad Child Adolesc Psychiatry.

[15] Ford T, Goodman R, Meltzer H (2003). The British Child and Adolescent Mental Health Survey 1999: the prevalence of DSM-IV disorders. J Am Acad Child Adolesc Psychiatry.

[16] Goodman R, Slobodskaya H, Knyazev G (2005). Russian child mental health—a cross-sectional study of prevalence and risk factors. Eur Child Adolesc Psychiatry.

[17] Mullick MS, Goodman R (2005). The prevalence of psychiatric disorders among 5–10 year olds in rural, urban and slum areas in Bangladesh: an exploratory study. Soc Psychiatry Psychiatr Epidemiol.

[18] Costello EJ, Mustillo S, Erkanli A, Keeler G, Angold A (2003). Prevalence and development of psychiatric disorders in childhood and adolescence. Arch Gen Psychiatry.

[19] Costello EJ, Compton SN, Keeler G, Angold A (2003). Relationships between poverty and psychopathology: a natural experiment. JAMA.

[20] Costello J, Egger H, Angold A (2005). 10-year research update review: the epidemiology of child and adolescent psychiatric disorders: I. methods and public health burden. J Am Acad Child Adolesc Psychiatry.

[21] van Oort FV, van der Ende J, Wadsworth ME, Verhulst FC, Achenbach TM (2011). Cross-national comparison of the link between socioeconomic status and emotional and behavioral problems in youths. Soc Psychiatry Psychiatr Epidemiol.

[22] Barnett K, Mercer SW, Norbury M, Watt G, Wyke S, Guthrie B (2012). Epidemiology of multimorbidity and implications for health care, research, and medical education: a cross-sectional study. Lancet.

[23] Canino G, Alegria M (2008). Psychiatric diagnosis—is it universal or relative to culture?. J Child Psychol Psychiatry.

[24] Wichstrøm L, Berg-Nielsen TS, Angold A, Egger HL, Solheim E, Sveen TH (2012). Prevalence of psychiatric disorders in preschoolers. J Child Psychol Psychiatry.

[25] Fundação de Economia e Estatistica do Rio Grande do Sul—Resumo Estatístico RS —Estado. http://www.fee.tche.br/sitefee/pt/content/resumo/pg_estado.php

[26] Pesquisa Nacional por Amostra de Domicílios—Síntese de Indicadores 2011. http://www.ibge.gov.br/home/estatistica/populacao/trabalhoerendimento/pnad2011/default_sintese.shtm

[27] Instituto Brasileiro de Geografia e Estatística (IBGE)—Indicadores. http://www.ibge.gov.br/home/mapa_site/mapa_site.php

[28] Barros AJ, da Silva dos Santos I, Victora CG, Albernaz EP, Domingues MR, Timm IK, Matijasevich A, Bertoldi AD, Barros FC (2006). The 2004 Pelotas birth cohort: methods and description. Rev Saude Publica.

[29] Santos IS, Barros AJ, Matijasevich A, Domingues MR, Barros FC, Victora CG (2011). Cohort Profile: the 2004 Pelotas (Brazil) birth cohort study. Int J Epidemiol.

[30] Goodman R, Ford T, Richards H, Gatward R, Meltzer H (2000). The Development and well-being Assessment: description and initial validation of an integrated assessment of child and adolescent psychopathology. J Child Psychol Psychiatry.

[31] WHO (1993). The ICD-10 Classification of mental and behavioral disorders: diagnostic criteria for research.

[32] APA (1994). Diagnostic and statistical manual of mental disorder (dsm-IV-fourth version).

[33] Ford T, Last A, Henley W, Norman S, Guglani S, Kelesidi K, Martin AM, Moran P, Latham-Cork H, Goodman R (2013). Can standardized diagnostic assessment be a useful adjunct to clinical assessment in child mental health services? A randomized controlled trial of disclosure of the development and well-being assessment to practitioners. Soc Psychiatry Psychiatr Epidemiol.

[34] Barros AJ, Victora CG (2005). A nationwide wealth score based on the 2000 Brazilian demographic census. Rev Saude Publica.

[35] Alyahri A, Goodman R (2008). The prevalence of DSM-IV psychiatric disorders among 7–10 year old Yemeni schoolchildren. Soc Psychiatry Psychiatr Epidemiol.

[36] Beesdo-Baum K, Knappe S (2012). Developmental epidemiology of anxiety disorders. Child Adolesc Psychiatric Clin N Am.

[37] Cohen P, Cohen J, Kasen S, Velez CN, Hartmark C, Johnson J, Rojas M, Brook J, Streuning EL (1993). An epidemiological study of disorders in late childhood and adolescence—I. Age-and gender-specific prevalence. J Child Psychol Psychiatry.

[38] Merikangas KR, He JP, Burstein M, Swanson SA, Avenevoli S, Cui L, Benjet C, Georgiades K, Swendsen J (2010). Lifetime prevalence of mental disorders in US adolescents: results from the National Comorbidity Survey Replication-Adolescent Supplement (NCS-A). J Am Acad Child Adolesc Psychiatry.

[39] Polanczyk G, de Lima MS, Horta BL, Biederman J, Rohde LA (2007). The worldwide prevalence of ADHD: a systematic review and metaregression analysis. Am J Psychiatry.

[40] Ullebø AK, Posserud MB, Heiervang E, Obel C, Gillberg C (2012). Prevalence of the ADHD phenotype in 7- to 9-year-old children: effects of informant, gender and non-participation. Soc Psychiatry Psychiatr Epidemiol.

[41] Kieling C, Kieling RR, Rohde LA, Frick PJ, Moffitt T, Nigg JT, Tannock R, Castellanos FX (2010). The age at onset of attention deficit hyperactivity disorder. Am J Psychiatry.

[42] Angold A, Erkanli A, Copeland W, Goodman R, Fisher PW, Costello J (2012). Psychiatric diagnostic interviews for children and adolescents: a comparative study. J Am Acad Child Adolesc Psychiatry.

[43] Ramtekkar UP, Reiersen AM, Todorov AA, Todd RD (2010). Sex and age differences in attention-deficit/hyperactivity disorder symptoms and diagnoses: implications for DSM-V and ICD-11. J Am Acad Child Adolesc Psychiatry.

[44] Reijneveld SA, Brugman E, Verhulst FC, Verloove-Vanhorick SP (2005). Area deprivation and child psychosocial problems—a national cross-sectional study among school-aged children. Soc Psychiatry Psychiatr Epidemiol.

[45] Costello EJ, Erkanli A, Copeland W, Angold A (2010). Association of family income supplements in adolescence with development of psychiatric and substance use disorders in adulthood among an American Indian population. JAMA.

[46] Fleitlich B, Goodman R (2001). Social factors associated with child mental health problems in Brazil: cross sectional survey. BMJ.

[47] Egger HL, Angold A (2006). Common emotional and behavioral disorders in preschool children: presentation, nosology, and epidemiology. J Child Psychol Psychiatry.

